# Mapping Dorsal and Ventral Caudate in Older Adults: Method and Validation

**DOI:** 10.3389/fnagi.2017.00091

**Published:** 2017-04-04

**Authors:** Haiqing Huang, Peter T. Nguyen, Nadine A. Schwab, Jared J. Tanner, Catherine C. Price, Mingzhou Ding

**Affiliations:** ^1^J. Crayton Pruitt Family Department of Biomedical Engineering, University of FloridaGainesville, FL, USA; ^2^Department of Clinical and Health Psychology, University of FloridaGainesville, FL, USA

**Keywords:** dorsal and ventral caudate, caudate function, functional connectivity, clustering analysis, resting state fMRI

## Abstract

The caudate nucleus plays important roles in cognition and affect. Depending on associated connectivity and function, the caudate can be further divided into dorsal and ventral aspects. Dorsal caudate, highly connected to dorsolateral prefrontal cortex (DLPFC), is implicated in executive function and working memory; ventral caudate, more interconnected with the limbic system, is implicated in affective functions such as pain processing. Clinically, certain brain disorders are known to differentially impact dorsal and ventral caudate. Thus, precise parcellation of caudate has both basic and clinical neuroscience significance. In young adults, past work has combined resting-state fMRI functional connectivity with clustering algorithms to define dorsal and ventral caudate. Whether the same approach is effective in older adults and how to validate the parcellation results have not been considered. We addressed these problems by obtaining resting-state fMRI data from 56 older non-demented adults (age: 69.07 ± 5.92 years and MOCA: 25.71 ± 2.46) along with a battery of cognitive and clinical assessments. Connectivity from each voxel of caudate to the rest of the brain was computed using cross correlation. Applying the K-means clustering algorithm to the connectivity patterns with *K* = 2 yielded two substructures within caudate, which agree well with previously reported dorsal and ventral divisions of caudate. Furthermore, dorsal-caudate-seeded functional connectivity was shown to be more strongly associated with working memory and fluid reasoning composite scores, whereas ventral-caudate-seeded functional connectivity more strongly associated with pain and fatigue severity. These results demonstrate that dorsal and ventral caudate can be reliably identified by combining resting-state fMRI and clustering algorithms in older adults.

## Introduction

The caudate is a gray matter subcortical nucleus that can be divided into dorsal and ventral aspects based on their connectivity and functions (Nakano et al., 2000; Robinson et al., 2012). The dorsal caudate, a component of the dorsal striatum, plays important roles in motor and cognitive functions (Choi et al., 2012; Robinson et al., 2012). By integrating spatial information and motor preparation, the dorsal caudate has been shown, among other functions, to be involved in spatial working memory (Levy et al., 1997; Postle and D'Esposito, 1999) and deductive reasoning (Rodriguez-Moreno and Hirsch, 2009). In contrast, the ventral caudate, a key component of the ventral striatum, is associated with reward processing (Knutson and Cooper, 2005; Haber and Knutson, 2010; Benningfield et al., 2014) and affective functions including the perception of pain (Jensen et al., 2003; Martikainen et al., 2015) and fatigue (Miller et al., 2014). In particular, clinical assessment of fatigue is significantly correlated with neural activity in the ventral striatum during hedonic reward tasks (Capuron et al., 2012).

In recent clinical studies, dorsal and ventral caudate show different vulnerability to diseases. The dorsal caudate appears to be more vulnerable to diseases that cause motor or cognitive impairments (e.g., Parkinson's disease), while the ventral caudate appears more disrupted in affective disorders. In the early stages of Parkinson's disease (PD), the dorsal caudate demonstrates dopamine depletion but the ventral caudate remains relatively intact (Grahn et al., 2008). Dorsal caudate atrophy has also been found to disrupt frontostriatal connections that are critical for executive function in a sample of patients with temporal lobe epilepsy (TLE) (Riley et al., 2011). On the other hand, deep brain stimulation (DBS) in the ventral caudate has had some success in the treatment of several affective disorders, including obsessive compulsive disorder (OCD) and major depression (Aouizerate et al., 2004). In Huntington's disease, there is significant caudate atrophy, with the ventral caudate showing more atrophy in the group of more severely affected patients (Kassubek et al., 2004).

To define dorsal and ventral caudate, previous neuroimaging studies primarily relied on anatomical features (Mawlawi et al., 2001; Postuma and Dagher, 2006). Postuma and Dagher defined the dorsal/ventral caudate boundary as an axial slice at z = 7 in the MNI template, and reported different co-activated patterns within dorsal and ventral striatum using meta-analysis (Postuma and Dagher, 2006). This caudate partition method has also been adopted in other neuroimaging studies, including mapping multiple distinct striatal circuits (Di Martino et al., 2008), correlating corticostriatal functional connectivity disturbances with OCD (Harrison et al., 2009), and relating corticostriatal functional connectivity alterations with depression (Kerestes et al., 2015). A more refined dorsal/ventral caudate boundary was proposed by Mawlawi et al. using anatomical landmarks in the human brain on MRI T1 images (Mawlawi et al., 2001).

Although anatomy-based caudate parcellation is simple and straightforward, it does not necessarily reflect connectivity (Mars et al., 2012). Given that connectivity is basis of function, parcellation methods based on anatomical features, while directly reflecting dorsal and ventral caudate structure, might not provide clear separation of functional characteristics. Researchers have begun to explore functional connectivity based parcellation of brain structures using resting state fMRI (Deen et al., 2011; Kahnt et al., 2012; Chang et al., 2013; Cao et al., 2014; Jung et al., 2014; Janssen et al., 2015; Eickhoff et al., 2016). Based on the functional connectivity profiles, each subdivision's function can be inferred from the brain areas to which it is connected (Kahnt et al., 2012; Jung et al., 2014; Janssen et al., 2015). In this line of work, validation of the parcellation results come primarily from task-based fMRI activations within each substructure (Deen et al., 2011; Chang et al., 2013; Eickhoff et al., 2016), behavioral validation has been lacking.

An additional shortcoming of the extant literature is that young healthy adults have been the main focus of caudate parcellation investigations. As brain structure and function change significantly over the lifespan (Hedden and Gabrieli, 2004; Grady, 2012; Samanez-Larkin and Knutson, 2015), to what extent the functional connectivity based parcellation applies to older adults with and without cognitive impairment has received less attention. The value of pursuing this question is demonstrated recently by Cao et al. (2014). Segregating anterior cingulate cortex (ACC) into dorsal/rostral subdivisions, Cao et al. showed that older adults have significantly different dorsal/rostral ACC seeded functional connectivity profiles compared to young adults; these differences might serve as part of the anatomical foundation for the cognitive and emotional alterations in the aging brain (Cao et al., 2014). Additionally, the caudate nucleus is known to undergo significant age-related volumetric loss along with memory related decline in old adults (Abedelahi et al., 2013; Bauer et al., 2015). This may pose challenges for analytical methods established mainly in the study of young adults.

To address the shortcomings identified above, we sought to parcellate the caudate nucleus in older adults using resting state fMRI. To validate the results, functional connectivity maps seeded in dorsal and ventral caudate were correlated with *a priori* selected neuropsychological measures tapping into dorsolateral caudate functions (i.e., working memory, reasoning) vs. ventral caudate functions (i.e., pain, fatigue).

## Materials and methods

### Participants

The University of Florida Institutional Review Board approved the experimental protocol. Fifty-six non-demented older adults who were participating in larger NIH-funded investigations and who had no history of neurological disease or head injury gave written informed consent for study participation. The research was conducted in accordance with the Declaration of Helsinki. All subjects were screened for possible risks or contraindications for MRI scanning.

### Neuropsychological data acquisition

Participants completed a comprehensive neuropsychological assessment as part of the larger NIH-funded investigations. Scores were standardized to published norms (Wechsler, 1999; Heaton and Psychological Assessment Resources Inc, 2004). For the present study, the primary variables of interest were standardized normative based composites of (1) *Working Memory*—based on Wechsler Memory Scale-III Digit Span Backward Span (longest span backward), Spatial Span Backward (total score), and Letter Number Sequencing (total score) and (2) *Reasoning*—based on Wechsler Abbreviated Scale of Intelligence (WASI) matrix reasoning subtest (total correct) and Tower Test (total achievement score).

Also in accordance with study hypotheses, we examined pain and fatigue. A total of 49 out of 56 participants completed a self-report brief pain inventory (BPI) (Cleeland and Ryan, 1994) and 44 out of 56 participants completed a self-report brief fatigue inventory (BFI) (Mendoza et al., 1999). Participants with higher BPI or BFI scores are reporting greater pain or fatigue, respectively. The final outcome variable for these measures included total raw score.

### MRI data acquisition

Functional and structural images were acquired on a Siemens MAGNETOM Verio 3T whole body MRI scanner with an 8-channel head coil. For resting state, 7.5 min of axial fMRI were recorded using a single-shot EPI sequence with the following parameters: field of view = 224 × 224 mm, matrix size = 64 × 64, TR = 2 s, TE = 30 ms, flip angle = 90°, slice thickness = 3.5 mm; 225 scans, and each volume consisting of 36 axial slices. T1 MPRAGE image of 176 sagittal slices were recorded with the following parameters: field of view = 256 × 256 mm, matrix size = 256 × 256, slice thickness = 1 mm.

### FMRI data preprocessing

Resting state fMRI images were preprocessed according to the following steps. The first five functional scans were discarded to eliminate transients. The remaining fMRI images were preprocessed using SPM (http://www.fil.ion.ucl.ac.uk/spm/). Slice timing correction was performed to compensate for acquisition delays across slices. Motion artifacts of timing corrected images were estimated and corrected by realigning all functional images to the first image. No participants were rejected due to excessive motion according to predetermined criteria (Long et al., 2008; Wylie et al., 2014). All the motion corrected functional images were co-registered to the T1 structural image, which were then normalized to the standard MNI152 T1 template, and resampled with 3 × 3 × 3 mm resolution. Functional images in the MNI template space were spatially smoothed with an 8 mm full width at half maximum (FWHM) isotropic Gaussian kernel. Resting state fMRI time series were then extracted from voxels within a gray matter brain mask after regressing out nine nuisance signals, including six movement variables and three averaged signals representing white matter, cerebrospinal fluid, and whole brain. These time series were bandpass filtered between 0.01 and 0.1 Hz with a finite impulse response (FIR) filter.

### Functional connectivity based parcellation of caudate

The bilateral caudate nucleus ROIs were defined according to the Automated Anatomical Labeling (AAL) atlas in the standard MNI template space. Cross correlation (CC) between each voxel of the caudate and all the other voxels in the gray matter mask of the entire brain was computed for each individual. The individual whole-brain correlation maps were converted to z-score maps by Fisher's z transformation. The z-score maps for each caudate voxel were averaged across subjects to obtain group-level whole-brain maps. The K-means clustering algorithm implemented in MATLAB was applied to the group-level whole-brain maps with *K* = 2. Dorsal and ventral caudate were defined according to the anatomical location of each cluster within caudate.

### Functional validation of caudate partition

For each participant, a caudate-cortex functional connectivity map was obtained by averaging the whole brain functional connectivity map from each caudate voxel across all caudate voxels. The resulting caudate-cortex maps were subjected to a one-sample *t*-test across subjects to obtain a group level caudate-cortex functional connectivity *t*-value map. Furthermore, a dorsal-caudate-cortex functional connectivity map and a ventral-caudate-cortex functional connectivity map for each participant were separately obtained by averaging the whole brain functional connectivity maps across all seed voxels within the dorsal caudate cluster and the ventral caudate cluster, respectively.

Whole-brain functional-connectivity-neuropsychological-assessments analysis were performed on dorsal-caudate-cortex functional connectivity maps and ventral-caudate-cortex functional connectivity maps by correlating the connectivity strength with the working memory index, the reasoning index, the pain score, and the fatigue score. Two regions of interest (ROIs) were selected to illustrate the association between dorsal-caudate-seeded and ventral-caudate-seeded connectivity and neuropsychological assessments. The ROI located in the left dorsolateral prefrontal cortex (lDLPFC), a region known for its importance in cognitive functioning (Barbey et al., 2013), contains voxels within a sphere of 5 mm in radius centered at MNI: [−42, 24, 45]. The ROI in the rostral anterior cingulate cortex (rACC), a region known for its importance in affective functioning (Vytal and Hamann, 2009; Cao et al., 2014), contains voxels within a sphere of 5 mm in radius centered at MNI: [6, 18, −6]. The center of each ROI corresponded to the peak voxel attaining local maximum *t*-value in the group level caudate-cortex functional connectivity *t*-value map. Dorsal and ventral caudate-lDLPFC functional connectivity were computed by averaging the cross correlation values across voxels in lDLPFC, and correlated with the working memory index and the reasoning index. Dorsal and ventral caudate-rACC functional connectivity was computed by averaging the cross correlation values across voxels in rACC, and correlated with the pain score and the fatigue score.

## Results

### Participant characteristics

As shown in Table 1, the cohort was comprised of 56 healthy older adults with mean age of 69.07 ± 5.92 years (age range: 56–83 years). The mean education duration was 16.23 ± 3.02 years (education duration range: 11–24 years). The mean score on a measure of global cognitive functioning, Montreal Cognitive Assessment (MoCA), was 25.71 ± 2.46. The scores on working memory (0.61 ± 0.72) and reasoning (0.90 ± 0.70) were within the average range. The mean pain score for the 49 participants with data was 26.65 ± 21.82 and the mean fatigue score for the 44 participants with data was 2.67 ± 1.95.

**Table 1:** **Participant characteristics**.

**Items**	**Number of participants**	**Range (min–max)**	**Mean ± *SD***
Age (years)	56	56–83	69.07 ± 5.92
Education (years)	56	11–24	16.23 ± 3.02
MOCA	56	21–30	25.71 ± 2.46
WMI	56	−0.89 to 2.55	0.61 ± 0.72
Reasoning	56	−0.64 to 2.47	0.90 ± 0.70
BPI	49	0–84	26.65 ± 21.82
BFI	44	0–7	2.67 ± 1.95

*MOCA, Montreal Cognitive Assessment; WMI, Working memory index; BPI, Brief pain inventory; BFI, Brief fatigue inventory*.

### Functional connectivity based parcellation of caudate

**Figure 1:**
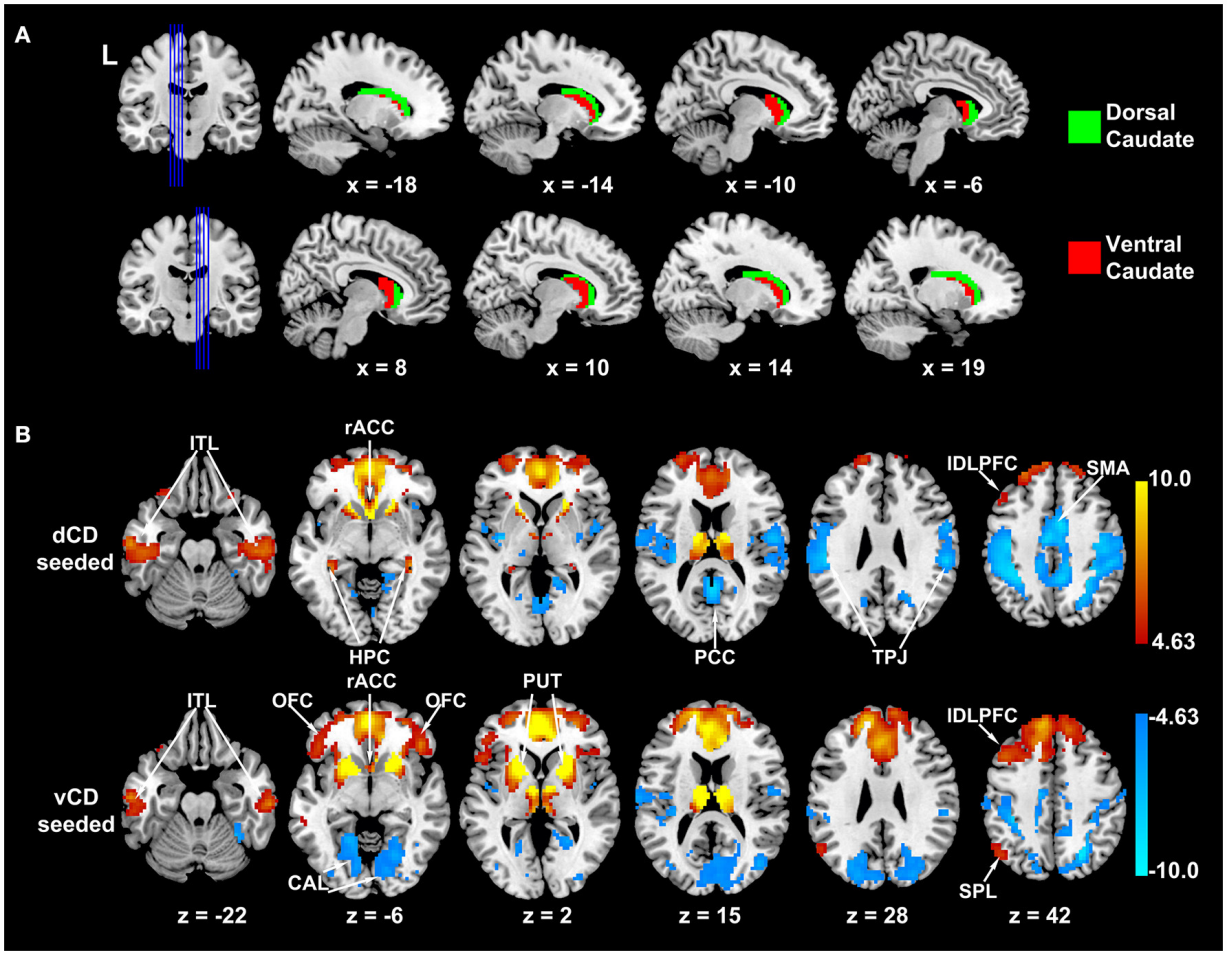
**Functional connectivity based parcellation of caudate in older adults**. **(A)** Dorsal (green) and ventral (red) caudate. **(B)** Dorsal and ventral caudate seeded functional connectivity maps (*p* < 0.0001 FDR corrected). Warm color indicates positive correlation; winter color indicates negative correlation. dCD, dorsal caudate; vCD, ventral caudate; ITL, inferior temporal lobule; rACC, rostral anterior cingulate cortex; HPC, hippocampus; PCC, posterior cingulate cortex; TPJ, temporoparietal junction; lDLPFC, left dorsolateral prefrontal cortex; SMA, supplementary motor area; OFC, orbitofrontal cortex; CAL, calcarine sulcus; PUT, putamen; SPL, superior parietal lobule.

Caudate-seeded whole-brain functional connectivity maps were computed. A K-means clustering analysis with K = 2 yielded two clusters. As shown in Figure 1A, one cluster (red) corresponds to the ventral and medial portion of caudate, while the other cluster (green) corresponds to the dorsal and lateral portion of caudate. Figure 1B shows dorsal-caudate-seeded and ventral-caudate-seeded whole-brain functional connectivity maps. The dorsal caudate subdivision was functionally connected with dorsolateral prefrontal cortex (DLPFC), supplementary motor area (SMA), rostral anterior cingulate cortex (rACC), posterior cingulate cortex (PCC), temporo-parietal junction (TPJ), and hippocampus (HPC), while the ventral caudate subdivision was associated with dorsal and rostral anterior cingulate cortex (ACC), dorsal lateral prefrontal cortex (DLPFC), superior parietal lobule (SPL), putamen (PUT), lateral orbital frontal cortex (OFC), and primary visual cortex.

### Functional validation of caudate subdivisions

To functionally validate the parcellation results, dorsal and ventral caudate seeded whole-brain functional connectivity values were correlated with the working memory index, the reasoning index, the pain score, and the fatigue score.

**Figure 2:**
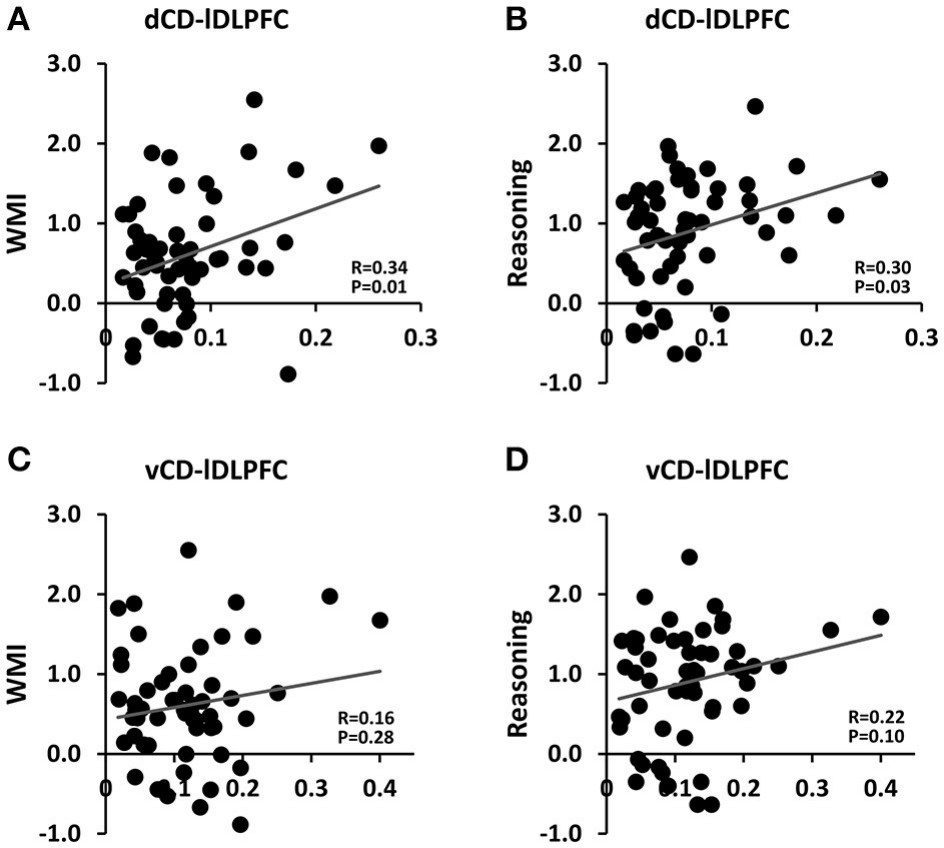
**Association between dorsal/ventral caudate-lDLPFC functional connectivity and working memory index (WMI)/reasoning index**. **(A)** Dorsal caudate-lDLPFC vs. WMI. **(B)** Dorsal caudate-lDLPFC vs. reasoning index. **(C)** Ventral caudate-lDLPFC vs. WMI. **(D)** Ventral caudate-lDLPFC vs. reasoning index.

**Figure 3:**
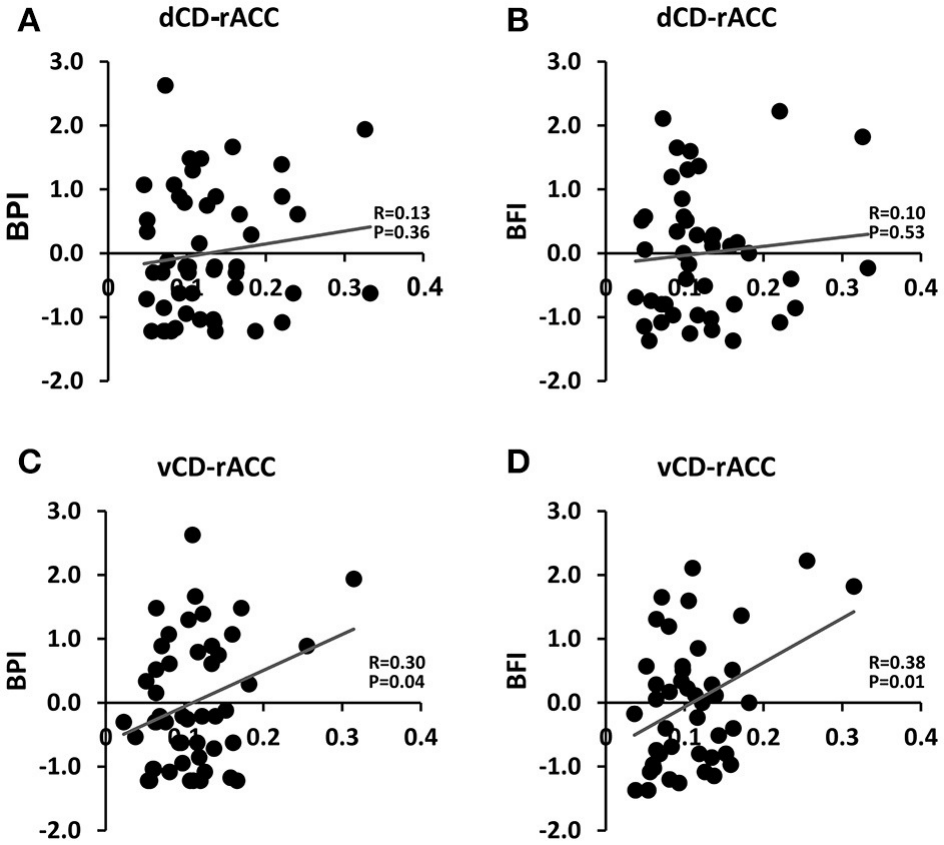
**Association between dorsal/ventral caudate-rACC functional connectivity and pain (BPI)/fatigue (BFI) scores. (A)** Dorsal caudate-rACC vs. BPI. **(B)** Dorsal caudate-rACC vs. BFI. **(C)** Ventral caudate-rACC vs. BPI. **(D)** Ventral caudate-rACC vs. BFI.

Two ROIs were selected first to illustrate the analysis. As shown in Figure 2, the dorsal caudate-left DLPFC functional connectivity were significantly positively correlated with the working memory index and the reasoning index, whereas the ventral caudate-left DLPFC functional connectivity were not. In contrast, as shown in Figure 3, the ventral caudate-rACC functional connectivity were significantly positively correlated with pain and fatigue, whereas the dorsal caudate-rACC functional connectivity were not. These results demonstrate the differential engagement of dorsal and ventral caudate in cognitive and affective functions.

**Figure 4:**
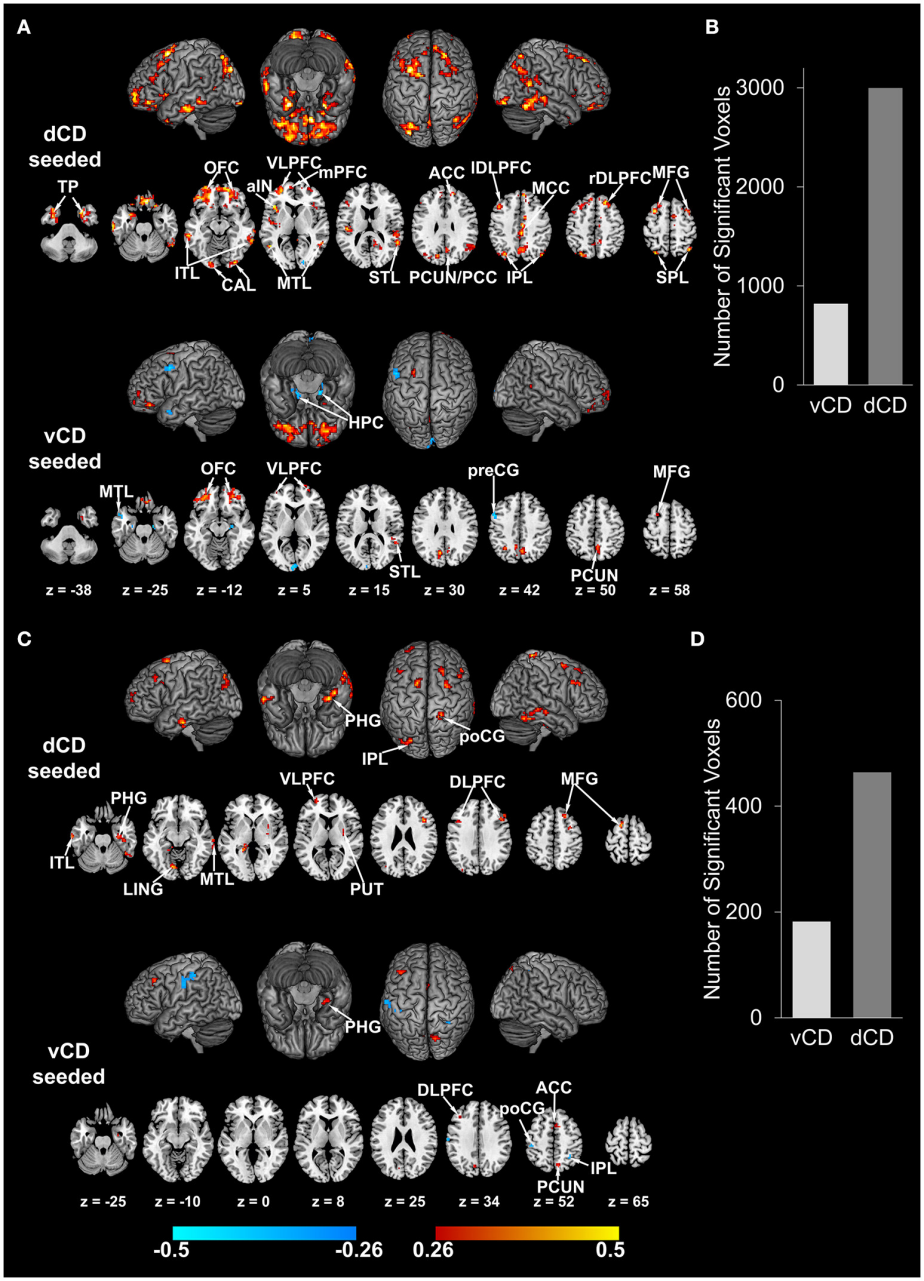
**Correlation maps between dorsal/ventral caudate-cortex functional connectivity and working memory index (WMI)/reasoning index (*p* < 0.05 FDR corrected)**. **(A)** Correlation maps between dorsal/ventral caudate seeded functional connectivity and WMI. Involved brain areas are summarized in Table 2. **(B)** Number of voxels significantly correlated with WMI for dorsal and ventral caudate. **(C)** Correlation maps between dorsal/ventral caudate seeded functional connectivity and reasoning index. Involved brain areas are summarized in Table 3. **(D)** Number of voxels significantly correlated with reasoning index for dorsal and ventral caudate. Warm color indicates positive correlation; winter color indicates negative correlation. TP, temporal pole; VLPFC, ventrolateral prefrontal cortex; mPFC, medial prefrontal cortex; aIN, anterior insula; ITL, inferior temporal lobe; MTL, middle temporal lobule; STL, superior temporal lobule; ACC, anterior cingulate cortex; PCUN/PCC, precuneus/posterior cingulate cortex; IPL, inferior parietal lobule; MCC, middle cingulate cortex; rDLPFC, right dorsolateral prefrontal cortex; MFG, middle frontal gyrus; preCG, precentral gyrus; PHG, para-hippocampal gyrus; poCG, post central gyrus; LING, lingual gyrus.

On the whole-brain level, as shown in Figure 4A and summarized in Table 2, there are 26 cortical areas whose connectivity strength with dorsal caudate are significantly positively correlated with working memory (positive correlation coefficient range: [0.26, 0.55]). In contrast, there are 12 cortical areas whose connectivity with ventral caudate correlated with working memory. Among these 12 significant correlations 6 were positive and 6 were negative (positive correlation coefficient range: [0.26, 0.44]; negative correlation coefficient range: [−0.26, −0.56]). In terms of the number of voxels the ratio is 2997 voxels (dorsal caudate) vs. 821 voxels (ventral caudate) (Figure 4B). For fluid reasoning (Figure 4C and Table 3), another measure of cognitive functioning, the results are similar. There are 13 cortical areas whose connectivity strength with dorsal caudate are significantly positively correlated with reasoning score (positive correlation coefficient range: [0.26, 0.45]). The number of cortical areas whose connectivity with ventral caudate was associated with the reasoning score was 6. Among these 6 significant correlations 4 were positive and 2 were negative (positive correlation coefficient range: [0.26, 0.37]; negative correlation coefficient range: [−0.26, −0.38]). In terms of the number of voxels the ratio was 464 voxels (dorsal caudate) vs. 182 voxels (ventral caudate) (Figure 4D).

**Table 2:** **Brain regions whose functional connectivity with dorsal/ventral caudate are significantly correlated with working memory index**.

**Anatomical regions**	**Dorsal caudate**				**Ventral caudate**			
	**MNI (X, Y, Z)**			**BA**	**MNI (X, Y, Z)**			**BA**
**FRONTAL**								
MFG	−21	21	60	8	−24	9	60	8
MFG	33	15	57	8				
SMA	6	−15	42	23				
mPFC	−9	63	0	10				
DLPFC	−42	21	42	9/44				
DLPFC	24	33	48	9				
VLPFC	−30	60	−3	11	−38	55	0	11/46
VLPFC	27	48	−9	11	31	60	2	11
preCG					−51	9	42	6
**TEMPORAL**								
STL	51	−30	21	48	51	−45	18	42
MTL	−63	−15	−21	21	−45	6	−24	20
MTL	57	−51	12	21				
ITL	−63	−51	−21	37				
ITL	63	−51	−18	37				
TP	−33	3	−39	36				
TP	24	12	−39	36				
HPC					−18	−9	−18	35
HPC					18	−12	−21	35
**PARIETAL**								
IPL	−30	−72	45	7				
IPL	39	−63	57	7				
PCUN	0	−57	36	7	3	−63	45	7
**OCCIPITAL**								
CAL	−21	−90	−15	18	0	−93	6	17
CAL	33	−90	−15	18				
**PARALIMBIC**								
OFC	−21	42	−12	11	−15	45	−15	11
OFC	21	42	−18	11	15	60	−6	11
ACC	−12	36	21	32				
MCC	6	−15	42	23				
aIN	−42	15	0	48				
aIN	42	18	9	48				

**Table 3:** **Brain regions whose functional connectivity with dorsal/ventral caudate are significantly correlated with reasoning**.

**Anatomical Regions**	**Dorsal caudate**				**Ventral caudate**			
	**MNI (X, Y, Z)**			**BA**	**MNI (X, Y, Z)**			**BA**
**FRONTAL**								
DLPFC	−42	24	36	44	−39	27	39	46
DLPFC	42	18	24	48				
VLPFC	−24	54	6	10				
MFG	−15	9	66	6				
MFG	27	6	60	8				
**TEMPORAL**								
MTL	69	−33	−6	21				
ITL	−63	−15	−27	20				
ITL	60	−54	−21	37				
PHG	36	−15	−30	20	30	−9	−27	36
**PARIETAL**								
PCUN					12	−72	57	7
IPL	−24	−78	45	7	33	−51	51	40
poCG	21	−42	75	1	−45	−30	48	2
**OCCIPITAL**								
LING	−12	−75	−9	18				
**PARALIMBIC**								
ACC					12	9	51	6/32
**SUBCORTICAL**								
PUT	30	3	0					

**Figure 5:**
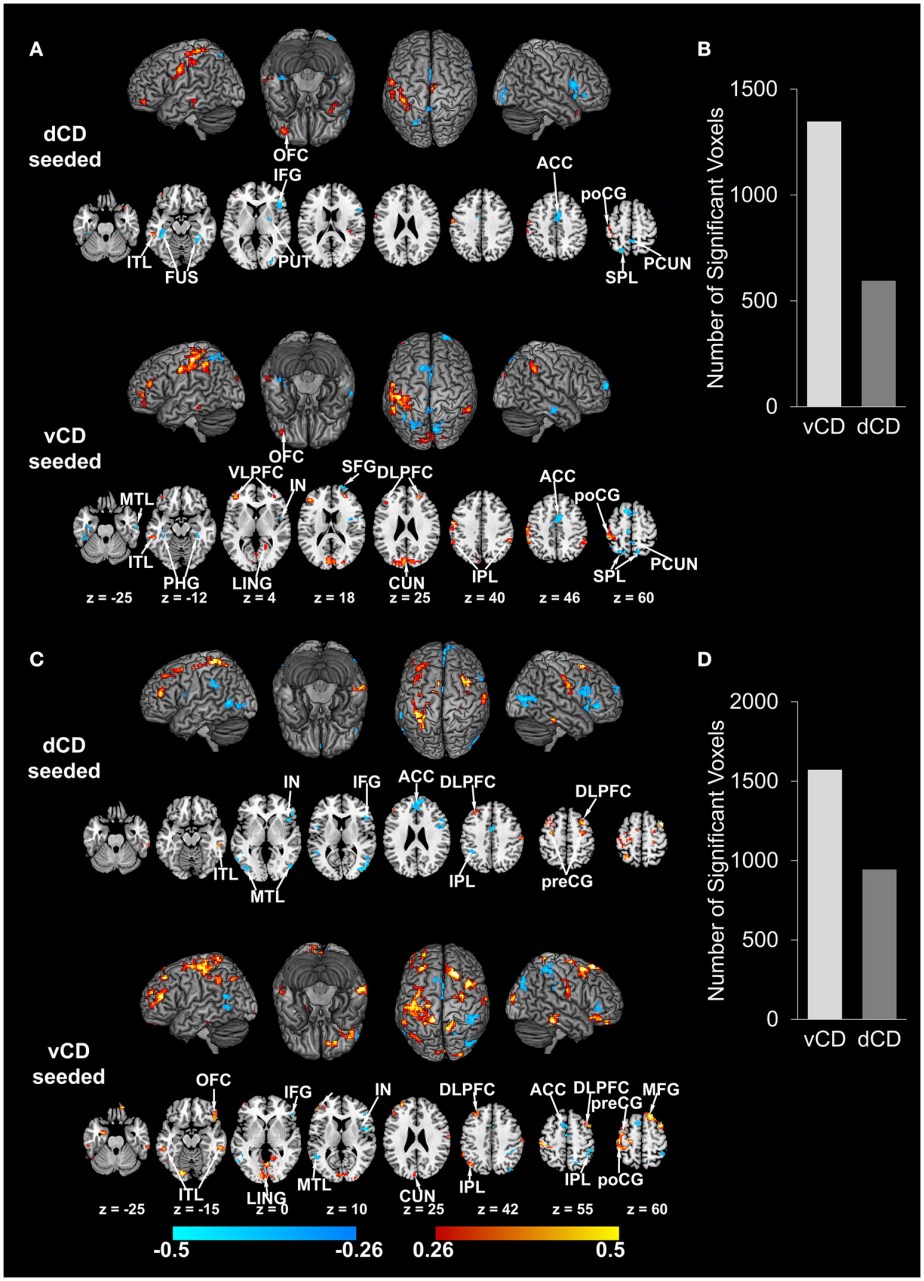
**Correlation maps between dorsal/ventral caudate-cortex functional connectivity and pain (BPI)/fatigue (BFI) (*p* < 0.05 FDR corrected)**. **(A)** Correlation maps between dorsal/ventral caudate seeded functional connectivity and BPI. Involved brain areas are summarized in Table 4. **(B)** Number of voxels significantly correlated with BPI for dorsal and ventral caudate. **(C)** Correlation maps between dorsal/ventral caudate seeded functional connectivity and BFI. Involved brain areas are summarized in Table 5. **(D**) Number of voxels significantly correlated with BFI for dorsal and ventral caudate. Warm color indicates positive correlation; winter color indicates negative correlation. FUS, fusiform; IFG, inferior frontal gyrus; IN, insula; SFG, superior frontal gyrus; CUN, cuneus.

For pain and fatigue, the situation is reversed. As shown in Figure 5A and Table 4, the number of cortical areas whose connectivity with dorsal and ventral caudate significantly correlated with pain was 10 vs. 20. Among the 10 dorsal caudate related correlations, 2 were positive and 8 were negative (positive correlation coefficient range: [0.28, 0.47]; negative correlation coefficient range: [−0.28, −0.45]). Among the 20 ventral caudate related correlations, 12 were positive and 8 were negative (positive correlation coefficient range: [0.28, 0.50]; negative correlation coefficient range: [−0.28, −0.49]). In terms of the number of voxels the ratio is 595 voxels (dorsal caudate) vs. 1347 voxels (ventral caudate) (Figure 5B). For fatigue, as shown in Figure 5C and Table 5, the number of cortical areas whose connectivity with dorsal caudate significantly correlated with fatigue was 10, whereas the number of cortical areas whose connectivity with ventral caudate significantly correlated with fatigue was 18. 5 out of the 10 dorsal caudate related correlations were positive and 5 were negative (positive correlation coefficient range: [0.30, 0.57]; negative correlation coefficient range: [−0.30, −0.49]). Among the 18 ventral caudate related correlations, 14 were positive and 4 were negative (positive correlation coefficient range: [0.30, 0.58]; negative correlation coefficient range: [−0.30, −0.48]). In terms of the number of voxels the ratio was 944 voxels (dorsal caudate) vs. 1572 voxels (ventral caudate) (Figure 5D).

**Table 4:** **Brain regions whose functional connectivity with dorsal/ventral caudate are significantly correlated with pain (BPI)**.

**Anatomical regions**	**Dorsal caudate**				**Ventral caudate**			
	**MNI (X, Y, Z)**			**BA**	**MNI (X, Y, Z)**			**BA**
**FRONTAL**								
DLPFC					−42	36	27	45
DLPFC					27	48	18	46
VLPFC					−42	45	3	45
VLPFC					39	48	3	47
SFG					27	66	18	10
IFG	48	30	3	45				
**TEMPORAL**								
MTL					60	−15	−21	20
ITL	−63	−30	−12	20	−54	−36	−15	20
PHG					−36	−33	−18	37
PHG					33	−33	−15	37
FUS	−36	−30	−18	20				
FUS	36	−36	−15	37				
**PARIETAL**								
PCUN	6	−48	60	5	6	−45	63	5
CUN					−3	−84	21	18
poCG	−42	−33	66	3	−48	−27	57	3
SPL	−21	−66	60	7	−24	−66	51	7
SPL					12	−66	57	7
IPL					−57	−42	48	40
IPL					48	−54	45	40
**OCCIPITAl**								
LING					24	−48	0	37
**PARALIMBIC**								
ACC	6	3	45	24	−9	18	60	6/32
OFC	−39	48	−12	47	−39	48	−12	47
IN					36	0	18	48
**Subcortical**								
PUT	27	−3	9					

**Table 5:** **Brain regions whose functional connectivity with dorsal/ventral caudate are significantly correlated with fatigue (BFI)**.

**Anatomical regions**	**Dorsal caudate**				**Ventral caudate**			
	**MNI (X, Y, Z)**			**BA**	**MNI (X, Y, Z)**			**BA**
**FRONTAL**								
MFG					18	30	63	8
DLPFC	−36	33	45	9	−30	36	45	9
DLPFC	39	12	60	9	51	15	48	9
VLPFC					−45	42	15	45
preCG	−24	−21	63	6	−24	−21	63	6
preCG	30	−6	54	6	18	−6	66	6
IFG	54	33	−3	45	51	33	3	45
**TEMPORAL**								
MTL	−48	−51	12	21	−54	−54	6	37
ITL					−48	−60	−9	37
ITL	60	−33	−21	20	60	−33	−21	20
**PARIETAL**								
CUN					−6	−90	30	19
IPL	−42	−42	39	40	−42	−66	42	39
IPL					45	−42	54	40
poCG					−45	−21	60	3
**OCCIPITAL**								
LING					−12	−96	−9	18
**PARALIMBIC**								
ACC	3	42	21	32	0	9	48	32
OFC					45	39	−18	47
IN	45	18	0	48	48	3	9	48

## Discussion

Combining resting-state fMRI connectivity and clustering analysis, we showed that it is possible to parcellate the caudate into two functional subdivisions: dorsal and ventral caudate, in older adults. The parcellation result is in line with previously proposed anatomical and functional organization of the caudate (Mawlawi et al., 2001; Postuma and Dagher, 2006; Di Martino et al., 2008). For validation we correlated dorsal caudate-seeded functional connectivity and ventral caudate-seeded functional connectivity with cognitive test scores and other clinical variables related to pain and fatigue. The results indicated that dorsal caudate-cortex functional connectivity was more strongly associated with cognitive functions including working memory and fluid reasoning, while ventral caudate-cortex functional connectivity was more strongly associated with affective functions including pain and fatigue, in agreement with theoretical expectations (Martinez et al., 2003; Choi et al., 2012; Robinson et al., 2012).

### Topographical organizations of caudate

Historically, the caudate was often divided into three portions: head, body, and tail (Hendelman, 2005). Alexander et al. proposed three frontostriatal circuits linking distinct cortical areas and different portions of caudate (Alexander et al., 1986). Specifically, the DLPFC projects to caudate body and dorsal part of caudate head, while lateral orbitofrontal cortex and anterior cingulate cortex were connected with ventromedial part of caudate. Because of their similar cellular infrastructure and structural connectivity, in recent human studies, caudate body, and caudate tail are combined into one anatomical region (Seger and Cincotta, 2005; Bernácer et al., 2007; Robinson et al., 2012; Seger et al., 2015). Applying probabilistic tractography on fronto-striatal connections, Leh et al. found that the anterior part of caudate (head) was strongly connected to ventral lateral prefrontal cortex (VLPFC), while dorsal lateral caudate (body/tail) was connected to DLPFC (Leh et al., 2007). In addition, Draganski et al. reported that the ventral medial prefrontal cortex (vMPFC) and orbitofrontal cortex (OFC) projected to the caudate head, while caudate body/tail preferentially connected with DLPFC, premotor, sensorimotor, and parietal cortex, an organization pattern referred to as “rostrocaudal gradient” of prefrontal connections in caudate (Draganski et al., 2008).

A different functional organization of caudate follows the subdivisions of striatum into dorsal and ventral striatum (Nakano et al., 2000; O'Doherty et al., 2004; Postuma and Dagher, 2006; Robinson et al., 2012; Jung et al., 2014). The intersection between dorsal striatum and caudate yielded the dorsal caudate, which was more strongly associated with cognitive and motor functions (Choi et al., 2012; Robinson et al., 2012). The ventral caudate, which is the intersection between caudate and ventral striatum (“limbic striatum”), was more strongly associated with affective functions (Martinez et al., 2003). In recent neuroimaging studies, dorsal and ventral caudate was anatomically separated either by a plane at z = 7 in MNI152 template (Postuma and Dagher, 2006; Di Martino et al., 2008; Harrison et al., 2009) or identified by anatomical landmarks (Mawlawi et al., 2001). Although these anatomical parcellation methods have been widely used, they have the shortcomings of not fully accounting for caudate functioning, since dorsal and ventral caudate are known to be functionally determined subdivisions. Given that connectivity is the basis for function, we adopted a functional connectivity based parcellation method using resting state fMRI data, which, when combined with clustering methods, was expected to yield caudate functional subregions. As shown in Figure 1, this method resulted in a dorsal caudate cluster, whose connectivity map included supplementary motor area (SMA), posterior cingulate cortex (PCC), temporo-parietal junction (TPJ), and hippocampus (HPC). The other cluster, which corresponded to the ventral portion of caudate, was functionally connected with anterior cingulate cortex (ACC), superior parietal lobule (SPL), putamen (PUT), lateral orbital frontal cortex (OFC), and primary visual cortex.

The above results in older adults can be compared to that in young adults. In young adults, summarizing co-activated brain areas in conjunction with dorsal/ventral striatum by meta-analysis, Postuma and Dagher found that the dorsal striatum co-activated with SMA, ACC, DLPFC, sensorimotor and motor cortex, while ventral striatum co-activated with medial temporal cortex, amygdala, and hippocampus (Postuma and Dagher, 2006). Using caudate-seeded resting state functional connectivity analysis, Di Martino et al. reported that ventral caudate primarily correlated with OFC while dorsal caudate with DLPFC (Di Martino et al., 2008). Applying functional connectivity based parcellation method on caudate with the number of clusters *K* = 2, Jung et al. found two subdivisions of dorsal/ventral caudate, and dorsal caudate was linked with DLPFC while ventral caudate was connected with ventral medial prefrontal cortex (vMPFC) (Jung et al., 2014). These results in young adults are highly similar to our results in older adults, despite potential age-related change in brain structure and function, demonstrating that the functional subdivision of caudate is preserved over a substantial age span.

### Validation of functional connectivity based parcellation

To validate the functional connectivity based parcellation of brain structures, past work has examined the functions of each subdivision either according to its functional connectivity profiles (Kahnt et al., 2012; Jung et al., 2014; Janssen et al., 2015) or by comparing it to task fMRI activation (Deen et al., 2011; Chang et al., 2013; Eickhoff et al., 2016). We took a step further by linking the dorsal/ventral-cortex functional connectivity with *a priori* selected cognitive test scores and clinical variables. At the ROI level, dorsal caudate-left DLPFC functional connectivity was significantly correlated with cognitive functions such as working memory and fluid reasoning, but ventral caudate-left DLPFC functional connectivity was not, as shown in Figure 2. On the other hand, ventral caudate-rACC functional connectivity was significantly correlated with affective functions such as pain and fatigue, but dorsal caudate-rACC was not, as shown in Figure 3. At the whole-brain level, dorsal caudate-seeded maps contained more areas (as well as more voxels) that were associated with cognitive functions, as shown in Figure 4 and Tables 2, 3, relative to affective functions. In contrast, ventral caudate-seeded maps contained more cortical areas (as well as more voxels) that were associated with affective functions, as shown in Figure 5 and Tables 4, 5, relative to cognitive functions. These findings are consistent with previous work on striatal subdivisions where dorsal and ventral portions of the striatum are associated with cognitive functions such as working memory, and affective functions, particularly pain and fatigue (Nakano et al., 2000; O'Doherty et al., 2004; Postuma and Dagher, 2006; Robinson et al., 2012). Specifically, ventral striatum, in addition to its strong involvement in emotional processing (Martinez et al., 2003; Postuma and Dagher, 2006; Jung et al., 2014) and reward, is also related to the symptoms of fatigue and pain (Jensen et al., 2003; Miller et al., 2014), which are known to have a strong affective component (Vuilleumier et al., 2001; Cardinal et al., 2002; Badgaiyan, 2010). Activation in ventral striatum by a reward task has been correlated with the clinical assessment of fatigue (Capuron et al., 2012), and dopamine neurotransmission change in ventral striatum was associated with chronic back pain (Martikainen et al., 2015). Dorsal striatum, connecting with dorsal prefrontal cortex, motor and sensorimotor cortex, was associated with cognitive and motor functions (Choi et al., 2012; Robinson et al., 2012; Jung et al., 2014). Previous resting state fMRI studies on caudate have reported similar findings that dorsal caudate was functionally connected to brain regions implicated in cognitive and motor control, while ventral caudate was shown to be more strongly connected to brain regions involved in affective processing (Di Martino et al., 2008).

The structure-function relationship is a long-standing question in neuroscience. The cohort of 56 older adults in this study afforded the opportunity to examine the structural characteristics of the areas within the caudate-related functional network by applying a single group voxel-based morphometry (VBM) analysis to the T1 images. As shown in the Supplementary Material, brain regions, whose volumes positively correlated with caudate volume, were consistent with those functionally connected to caudate, including bilateral putamen, bilateral anterior insula, left DLPFC, left fusiform, calcarine, anterior cingulate cortex, and right superior frontal gyrus. This suggested that there is a close relationship between functional connectivity patterns and brain volume changes.

### Functional relation between caudate and insula

Further insights can be gained into the functional subdivisions of the caudate by examining their relation with the insula which is another functionally and cytoarchitectonically diverse region (Kurth et al., 2010; Deen et al., 2011). Previous neuroimaging studies has revealed separate functional roles for distinct insular subregions (Kurth et al., 2010; Chang et al., 2013; Klein et al., 2013; Christopher et al., 2014). Specifically, the dorsal and ventral anterior insula was respectively related to cognitive and social-emotional functions (Kurth et al., 2010; Klein et al., 2013; Christopher et al., 2014), while the posterior insula was associated with somatosensory and autonomic processing, including interoception, somatosensation, and pain (Kurth et al., 2010; Klein et al., 2013). Anterior insula is activated by a broad range of cognitive tasks. Posterior insula is shown to be activated by painful stimuli (Carlsson et al., 2006; Maihöfner et al., 2006; Wunderlich et al., 2011). In Parkinson's disease dopaminergic and serotonergic dysfunction in middle posterior insula contributed to the fatigue symptom (Pavese et al., 2010). Our caudate-insula functional connectivity results are in agreement with these imaging studies. As shown in Figures 4, 5, anterior insula-dorsal caudate functional connectivity was significantly correlated with working memory, while middle posterior insula-ventral caudate functional connectivity was significantly correlated with pain and fatigue. These results demonstrated that subdivisions underlying similar functions in two different brain structures are connected to support those functions. Additional significant correlations between dorsal caudate-anterior insula functional connectivity and fatigue reflect the multifaceted nature of fatigue. Anterior insula was found activated immediately prior to the termination of fatiguing isometric handgrip contractions, suggesting its role in interpreting sense of effort and reward (Hilty et al., 2011a). Furthermore, the fact that communication between anterior insula and motor cortex was enhanced during fatiguing exercise indicated that anterior insula can integrate and evaluate sensory information from the periphery (Hilty et al., 2011b; Noakes, 2012). Theoretically, the sensation of fatigue has been proposed as conscious awareness of changes in subconscious homeostatic control system, which led to changes in brain activity and was perceived by consciousness-producing structures in the brain such as the insula (Gibson et al., 2003).

### Determining the number of clusters

In the K-means clustering algorithm used in the functional connectivity based parcellation methods, the optimal number of clusters K, corresponding to the number of subdivisions to be segregated, plays an important role (Eickhoff et al., 2015). For a given brain area to be segregated, there are two ways to determine the optimal number of clusters. One way is to set the optimal number according to the predefined number of subregions established in the literature (Chang et al., 2013). Another way is to compute a metric called “variation of information” (VI) as a function of number of clusters K by estimating the stability of cluster solutions (Kahnt et al., 2012; Jung et al., 2014). The optimal number of clusters is given as the smallest K for which stability (i.e., VI) does not substantially decrease relative to K–1 (Kahnt et al., 2012). For connectivity based parcellation on human caudate, Jung et al. have explored the number of clusters in both ways above, segregating caudate into specific (*K* = 2 and 3) or optimal (*K* = 9) number of subdivisions (Jung et al., 2014). When setting the number of clusters to *K* = 2, Jung et al. reported that caudate was segregated into two portions: ventral anterior caudate and dorsal posterior caudate (Jung et al., 2014), which were in line with the dorsal and ventral functional organization of the striatum. Based on the previous literature, especially on *a priori* hypothesis about the number of caudate subregions in functional organization, we therefore defined the number of clusters as *K* = 2 in this study.

### Summary and outlook

In this study we demonstrated the effectiveness of a functional connectivity based method to parcellate the caudate nucleus in older adults. The differential functionality of dorsal and ventral caudate were distinguished by relating dorsal and ventral caudate-cortex functional connectivity with specific cognitive and clinical assessments. Future studies should examine dorsal-ventral caudate nucleus segmentation in patient groups. There is ample evidence showing that the caudate nucleus is associate with cognitive impairment and potential dementia development, e.g., in Parkinson's disease and Huntington's disease. In non-demented Parkinson's disease, caudate volume was found to be positively correlated with processing speed (Price et al., 2016). In pre-manifest and early Huntington's disease, caudate volume was negatively correlated with disease burden score (Novak et al., 2014). Examination of these structures from the point of view of functional connectivity is expected to yield fresh insights that might lead to better diagnosis tools and intervention strategies.

## Author contributions

HH performed the experiment, analyzed image data, performed statistical results, and drafted the manuscript. PN performed the experiment and collected the data. NS, JT, and CP performed the experiment, collected the data, and revised the manuscript. CP and MD designed the study and gave critical comments on the manuscript.

### Conflict of interest statement

The authors declare that the research was conducted in the absence of any commercial or financial relationships that could be construed as a potential conflict of interest.
