# Supplementary material for: Mapping Dorsal and Ventral Caudate in Older Adults: Method and Validation
Source: Front Aging Neurosci. 2017 Apr 4;9:91. doi: 10.3389/fnagi.2017.00091 (PMC5378713; doi:10.3389/fnagi.2017.00091)
Supplement: Supplementary file 1 [file Presentation1.PDF]

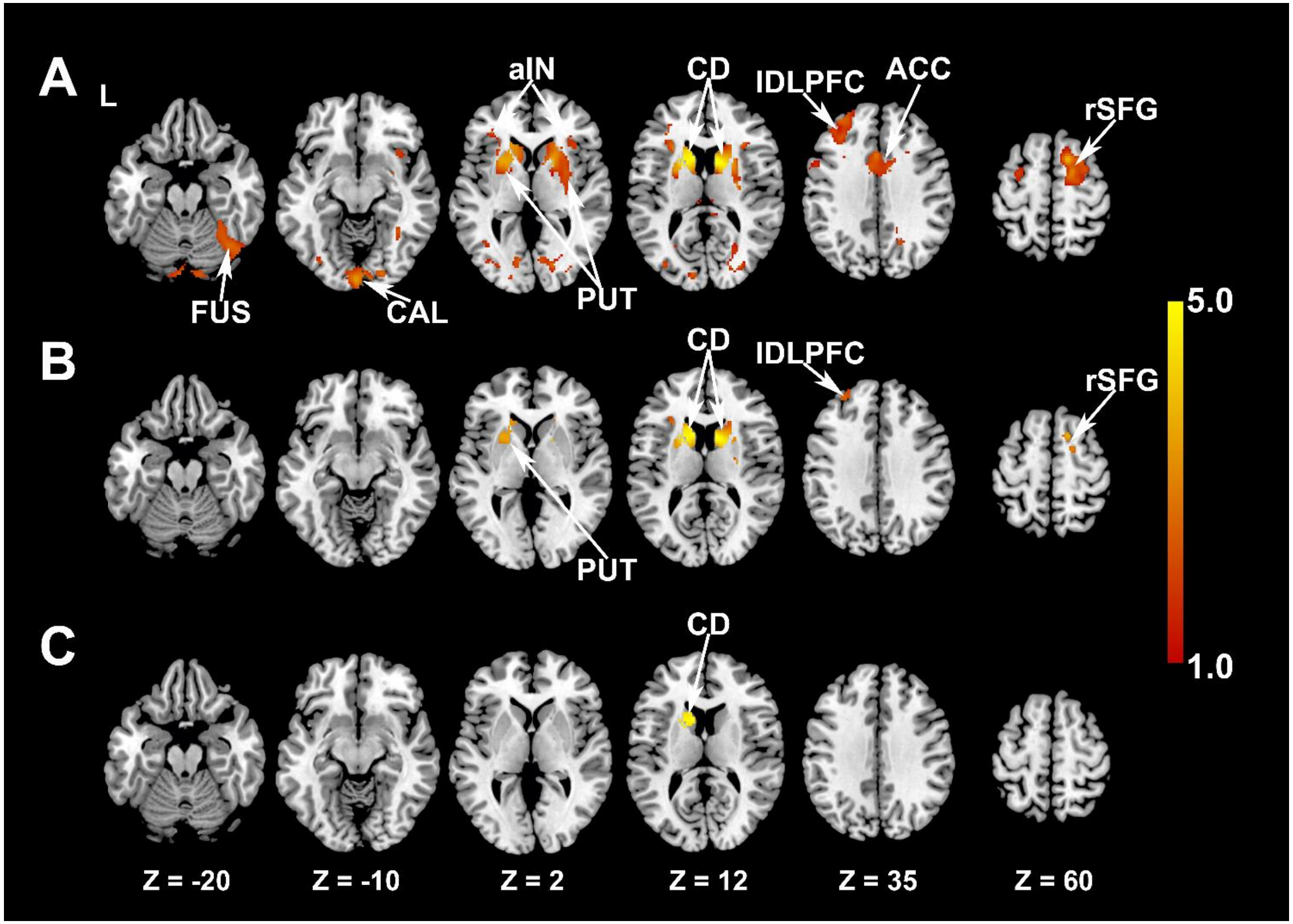

Supplementary Figure 1. Brain maps showing areas whose GM volume positively correlated with caudate GM volume. **A**) Uncorrected P<0.05, **B**) Uncorrected P<0.01 and **C**) TFCE corrected P<0.05. Color brightness indicates t-value of the regression analysis. FUS: fusiform; CAL: calcarine; aIN: anterior insula; PUT: putamen; CD: caudate; lDLPFC: left dorsolateral prefrontal cortex; ACC: anterior cingulate cortex; rSFG: right superior frontal gyrus.
